# Public Involvement in the Evaluation of Local Government Public Health Interventions in the UK: Lessons From PHIRST Insight

**DOI:** 10.1111/hex.70618

**Published:** 2026-02-26

**Authors:** Georgina Kathryn Wort, Hannah Robinson, Chloe Forte, Hannah Littlecott, Jemma Hawkins, Rona Campbell, Patricia Jessiman

**Affiliations:** ^1^ Department of Population Health Sciences, Bristol Medical School University of Bristol Bristol England, UK; ^2^ DECIPHer, School of Social Sciences, Social Sciences Research Park Cardiff University Cardiff Wales UK

**Keywords:** evaluation, interventions, local government, PCIEP, PPIE, public health

## Abstract

**Introduction:**

The value of public involvement in research is increasingly recognised, bringing specific lived experience to inform research from the outset. Despite known benefits, barriers to meaningful public involvement remain and further studies are needed to understand how it can be embedded throughout public health research evaluations across diverse contexts.

**Aim:**

To understand how public involvement has been embedded across 10 evaluations of local government public health initiatives.

**Methods:**

This study focuses on public involvement in 10 studies of local government public health interventions undertaken by an academic team funded through the NIHR PHIRST scheme. Semi‐structured interviews were conducted with three groups: public partners (*n* = 12), local government partners (*n* = 13) and members of the academic research team (*n* = 12), to share their experiences and reflections on embedding public involvement within this research context.

**Results:**

Several challenges to embedding public involvement were identified including studies' geographical locations, tight timelines, clarifying the role and expectations of public contributors, as well as the provision of training and support. Participants also noted how practices had developed over the 5‐year funding period. All participant groups highlighted the positive impact of public involvement, not only on individual studies, but also the reciprocal benefits to public partners and changes to longer‐term practices within local governments. However, awareness of the impact of public involvement could be improved by more formalised recording and feedback mechanisms.

**Conclusion:**

Our findings reinforce the importance of continued reflection, resource investment and structured processes to ensure meaningful public involvement is implemented, developed, evaluated and sustained over time.

**Public Contribution:**

Our public partners, Christina Stokes, Sian Harding, Rashmi Kumar and Barbara Harrington, made significant contributions to the study design and data collection materials (developing and refining the topic guides) developed for this study.

**Abbreviations**
PCIEP: Public and Community Involvement, Engagement and Participation
PPIE: Patient and Public Involvement and Engagement

## 1 Introduction

It is widely recognised that involving patients, service users and the public in research is important in achieving research outputs that are relevant, high‐quality and support sustainable improvement in health and social care [1, 2, 3]. Public involvement in health research is well established internationally [1, 4], and in the UK, the national funding agency for science and research is a signatory to the Shared Commitment to Public Involvement in health and social care research [5]. The UK National Institute of Health and Care Research (NIHR) now requires researchers to present a plan for public involvement, or if they do not plan to involve members of the public, to provide an explanation why [6, 7].

There are several arguments for public involvement in research. From a quality perspective, active involvement enables public contributors' unique knowledge and their specific lived experiences to inform the research from the outset [8]. Early involvement provides an opportunity for contributors to identify research questions and address aspects of the topic that researchers may have missed [9]. The specialist knowledge gained through experiencing particular health conditions, living in a specific locality, or using services can mean public contributors enhance the relevance of research questions and improve the quality of recruitment materials and data collection tools, bring unique insights to data interpretation and boost the dissemination of findings to larger and more diverse audiences [1, 10]. There is also a democratic argument; public money funds a large proportion of health research in the UK, and therefore, members of the public have a right to influence what research is supported and how it is conducted [8, 11]. In the UK, there is a legal duty for NHS England to involve patients and the public in commissioning and decision‐making around health services [12, 13]. Public involvement may also be used as a means of building support for research activity, involving public contributors in raising awareness, funds and contributing personal data to research [10].

Whilst the use of public involvement has been increasingly embraced by researchers, it is arguable that the barriers to doing so effectively have persisted, largely unchanged, over a 20‐year period [7]. A notable challenge is balancing meaningful public involvement with the time and resources available to researchers, and for them to deliver (or source) appropriate training for public partners to be fully involved, avoiding tokenism [1, 10]. Exploring the labour required by researchers to facilitate public involvement, including by public involvement coordinators, is important, as this is a typically under‐investigated group, often lacking recognition [14]. There are additional challenges associated with public involvement, such as power imbalances, potential conflicts and disagreements about roles and the focus of interest [8, 11]. Involvement may disempower public contributors if their input is not valued or acknowledged [11, 15]. Despite challenges identified, authors have reported that the disadvantages of involvement tend to be overlooked and underreported [11, 16].

Understanding what facilitates effective involvement and building on previous findings is important to advance the field. Public contributors are more likely to engage in studies which align with their own priorities, so it is also important that there is a shared understanding of the moral and methodological purposes of public involvement [17, 18]. There are important considerations for doing it well, such as allocating sufficient time and resources; developing a clear recruitment and training plan; fostering positive working relationships; valuing, acknowledging and rewarding public involvement [1, 15]. It is also important to be transparent about potential roles and responsibilities as early as possible [19] and to ensure there are opportunities for reflection and feedback throughout the study, both to public contributors and researchers [7, 9].

The assumption that the public wants to be involved in research has been questioned, as well as the capacity and need for ‘research literacy’ [11]. This leads to a ‘paradox of representation’, public members can be seen as either lacking the skills and knowledge to meaningfully contribute to a research study, or conversely, too experienced and knowledgeable to be representative of the ‘average' patient or service user [20]. This paradox was also observed in a study of public involvement in healthcare professional education when comparing the perspectives of both public contributors and academics [21].

In their review of public involvement in one applied health research partnership (a Collaboration for Leadership in Applied Health Research and Care), Knowles et al. [22] found that the representativeness of public partners was used by researchers to both argue for and against accepting their contributions. Where public input supported research activity, it was described by researchers as representative of ‘the patient view'. Where public members challenged the researcher's plans, their lack of representation was used as a defence [22].

In public health research, with its focus on improving population health and reducing health inequalities, the target population of interest is frequently those with poorer health outcomes than the general population, for example, people on low incomes, with disabilities and/or from minority ethnic groups. Ensuring diversity and equity of opportunity has been highlighted as an important component of public involvement and guidance for UK research more broadly [17, 19]. The NIHR has set out characteristics common to these ‘under‐served’ groups, including experiencing a higher healthcare burden, and lower inclusion in health research than the general population [3, 23]. In their study of the involvement of under‐served communities in public health research, McGrath et al. [3] highlight the importance of investing enough time to build relationships and establish trust between researchers and public contributors, clear and transparent communication about the likely impact of the research, flexible recruitment and engagement strategies and appropriate training and remuneration.

Previous research has predominantly discussed the challenges of embedding public involvement from a researcher or public member perspective in isolation, but has rarely combined both viewpoints from the same study [14]. This study looks at the experience of both researchers and public partners involved in applied evaluations of public health interventions commissioned by local government in the UK.

Public Health Intervention Responsive Studies Teams (PHIRST) are funded by the NIHR to provide timely and accessible evaluations of localised public health interventions [24]. The scheme was launched in 2020 with 4 academic research teams and expanded to 10 teams across the UK by 2025. A key driver behind the launch of PHIRST was recognition that whilst local governments play an important part in public health improvement, they are not always able to access the research and evaluation support to establish what works, and build the evidence base for local authority public health practice [25]. Following allocation of studies by a central NIHR programme team, PHIRST teams have 6 weeks to work with local partners to determine if the intervention is evaluable, and a further 6 weeks to develop a study protocol. This makes for a challenging context in which to embed public involvement into PHIRST studies. PHIRST teams can build on public involvement activity which their local government partners may have already undertaken, although in practice this may be limited. Local governments undertake consultations with their communities on many matters, but these can be qualitatively different from the public involvement typically sought in a PHIRST evaluation. PHIRST Insight, a collaboration between researchers from the Universities of Bristol and Cardiff, produced evaluations of 10 local government public health interventions between 2020 and 2025, details of which can be found in Table 1 and online [26]. Each of the 10 studies included involvement with members of the public local to the study. We focus here on involvement, undertaking research with members of the public (referred to within PHIRST Insight and from here in the paper as public partners), rather than engagement, where information about a piece of research is provided to or discussed with members of the public. Whilst there was variation across studies, the types of activities that public partners were involved in included contributing to study management groups, co‐producing protocols, the collection and analysis of qualitative data and the dissemination of findings, including co‐producing study outputs.

**Table 1:** Projects evaluated within PHIRST Insight and the number of PCIEP partners and researchers involved in the study.

Project topic	Location	Demographic	Number of PCIEP partners involved with study
Universal free school meal provision	London Borough of Hammersmith and Fulham	Secondary schools	1 PCIEP school
Mindset educational programme/intervention	Scotland	Primary and secondary schools	1 PCIEP school
Food train Eat well, Age well, nutritional checklist	Scottish Borders	Older adults	3
Community kitchen scheme	Leicestershire County Council	All local residents	6
Mental health community project	Haringey Council, London	Seven priority groups: those who belong to a minority ethnic group, whose first language is not English, low‐income households, older people, people with autism or learning disabilities, homeless and rough sleepers, young people not in education, employment or training	6
Active travel in market towns	Oxfordshire County Council	All local residents	1
NHS digital health check service	Southwark Council, London	All local residents	4
Falls Management Exercise (FaME) programme	Lincolnshire County Council	Older adults	4
Healthier advertising	Cardiff and Vale of Glamorgan	Secondary schools	1 PCIEP school and 1 public involvement representative
Planning development guidance	Brent Council	All local residents	12
Management Group			5
Advisory Group			2

At the outset of PHIRST Insight, a small number of researchers and public partners drafted a Public and Community Involvement, Engagement and Participation (PCIEP) strategy, which was discussed in detail by the team's internal Management Group and independent Advisory Board (both of which include public partners as group members) and revised before being approved and adopted. It was then reviewed annually with the Advisory Board and revised where necessary. The aim of the study reported here was to understand: how public involvement has been embedded within PHIRST Insight studies in practice; the challenges of embedding public involvement; its impact, and what lessons can be learnt to inform future public involvement practice.

## 2 Materials and Methods

This study is part of the NIHR PHIRST initiative and focuses on the 10 studies undertaken by PHIRST Insight [27]. The researchers conducting the interviews with public partners and academic researchers had not been involved in the same PHIRST Insight studies as the interviewees, and the lead author GW had had no involvement in PHIRST prior to commencing this work. Data collection occurred between May 2024 and June 2025. Prior to all data collection activities, participants were sent a detailed online information sheet detailing the aims of the study, funding, information about confidentiality, use of data and reporting. Online informed consent was provided by all participants, completing a form and confirmed verbally at the start of each interview.

### 2.1 Participants

Participants were purposively sampled from three groups: members of the PHIRST Insight academic research team, local government partners (including some third‐sector partners) and public partners. This latter group comprised members of the public recruited locally to support individual PHIRST studies, and those involved in Insight's Advisory Board (which gives strategic advice across the PHIRST Insight programme) and Management Group (which manages the day‐to‐day running of studies).

Public and local government partners were invited to participate in interviews to share their views and experiences of being involved in PHIRST Insight. Those who were interested in taking part were then directed to an online participant information sheet and consent form (hosted on JISC online survey for public partners and signed digitally for local government partners). An additional demographic questionnaire (hosted on JISC online survey) was sent to public partners to anonymously complete. Public partners were compensated for their time, receiving either a £25 payment or a voucher.

Academic researchers from the University of Bristol and Cardiff University who worked on PHIRST Insight studies, as well as the academic programme leads, were also invited to participate in semi‐structured interviews. Prior to interviews, consent was obtained using an online survey platform (JISC) and confirmed verbally at the start of each interview.

### 2.2 Data Collection

Data collection via semi‐structured interview was conducted either online through Microsoft Teams or by telephone and audio recorded. Interviews with public partners and academic researchers were conducted by GW and HR. Interviews with local government partners were conducted by PJ, CF and JH. The topic guide for public partners was developed in partnership with public members of PHIRST Insight's Advisory Board and Management group (some of whom were also subsequently interviewed for the study). Questions were open‐ended, allowing flexibility to explore individuals' responses in depth. Topic guides for all three participant groups are included as supporting materials.

**Figure 1:**
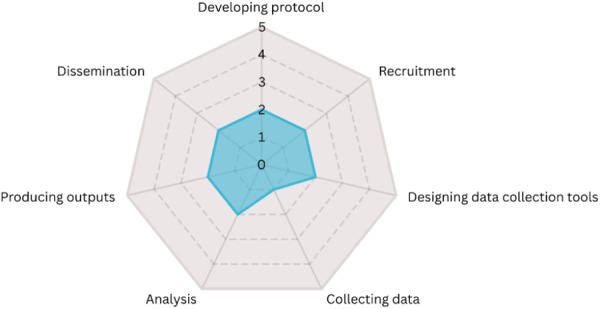
Example of a radar plot used to encourage participants to reflect on the level of PCIEP involvement [13].

**Figure 2:**
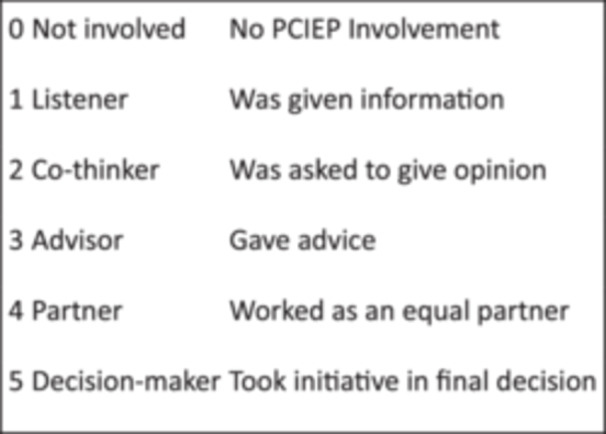
Levels of public involvement used within interviews, aligned with the Involvement Matrix [8].

During the interviews with both public partners and researchers involved in individual studies (rather than advisory or management groups), a radar plot (example in Figure 1) was used as a participatory tool to encourage individuals to reflect on the level of public involvement in the study. This was in line with the Involvement Matrix [8] used to record and discuss public involvement throughout the different stages of the research cycle (developing the protocol, recruiting, designing data collection tools, collecting data, analysing data, producing outputs and disseminating findings). Figure 2 details the different levels and was used to prompt both public partners and researchers, who were asked to rate their perception of public partner involvement from 0 to 5: 0 being no involvement and 5 taking the initiative in final decision making. The radar plot was used to facilitate the discussion, rather than as a formal data collection tool.

### 2.3 Data Analysis

Interviews were transcribed verbatim and anonymised, with false starts and irrelevancies removed, before the Framework method of thematic analysis was applied [28]. GW and HR developed a draft thematic framework based on the research questions, interview templates and having familiarised themselves with the transcripts. The draft framework was then used to inform an initial coding stage, before being further refined by GW, HR and PJ. A short definition of each theme was developed to describe the data captured. This framework was then shared with the whole research team and further refined until confident that it could be used to appropriately encompass all the data within the transcripts. Separate frameworks were created for researcher, public partner and local government partner interviews. The transcripts were charted by GW, HR and PJ, consistency and agreement were achieved by at least two researchers comparing the application of subthemes with a subset of the transcripts. Matrices were created with verbatim and summarised data from transcripts, showing data from every respondent within each subtheme, providing an accessible overview of the qualitative dataset. A summary of data was then used to inform the next stage of analysis, exploring patterns and associations between themes within the data, informing subsequent analysis and reporting. No public contributors were involved in the analysis or reporting of this study.

## 3 Results

A total of 12 public partners, 12 academic researchers and 13 local government partners were interviewed. Table 2 shows the number of respondents interviewed involved in each of the 10 studies and in the PHIRST management or advisory group. While a researcher and local government staff member involved in each of the 10 studies were interviewed, we were unable to recruit public partners from all studies. This was due to public partners involved in early Insight studies being difficult to recontact, or young people involved in school‐based studies for whom we did not have consent to recontact. We recruited 8 of the total 37 adults who were public partners across the 10 individual studies, and 4 of 7 public members (or previous members) of the Insight management or advisory group. Public partners' demographics are reported in Table 3 (demographic data were not recorded for researchers or local government respondents).

**Table 2:** Study participants' involvement in PHIRST projects, and the PHIRST insight management and advisory groups.

PHIRST study/management/advisory group	No. of researchers interviewed^a^	No. of public partners interviewed	No. of local government staff interviewed
Universal free school meal provision	2		1
Mindset educational programme/intervention	2		1
Food train eat well, age well, nutritional checklist	1		2
Community kitchen scheme	3	1	1
Mental health community project	2		1
Active travel in market towns	2		1
NHS digital health check service	2		1
Falls Management Exercise (FaME) programme	1	2	2
Healthier advertising	2	1	1
Planning development guidance	2	4	2
Management Group	2	2	
Advisory Group	2	2	

^a^ Some researchers were involved in multiple studies.

**Table 3:** Demographic information of public partners involved in this study.

Demographic information	Number (%) involved
Gender	
Woman	8 (67%)
Man	4 (33%)
Age	
31–40	1 (8%)
51–60	3 (25%)
61–70	7 (58%)
71+	1 (8%)
Highest level of education	
Higher or secondary education	3 (25%)
University degree	5 (42%)
Post‐graduate degree	2 (17%)
Prefer not to say	2 (17%)
Employment status	
Employment for wages	1 (8%)
Self‐employed	1 (8%)
Homemaker and/or carer	1 (8%)
Prefer not to say	1 (8%)
Retired	8 (67%)
Ethnicity	
White British	9 (75%)
Indian	1 (8%)
Arab	1 (8%)
Mixed Asian from Africa	1 (8%)

### 3.1 Unique Challenges

The nature of PHIRST studies and partnering with local government brought unique challenges to the involvement of members of the public, with researchers recognising the importance of adapting recruitment methods, the roles and tasks of public members (e.g., development of research questions and protocols, development of participant recruitment and data tools, co‐producing study outputs) depending on the aims and context of each study. PHIRST studies are not allocated based on the geographical location of PHIRST teams; researchers often conduct studies in unfamiliar locations and have to develop an understanding of the local context, including socio‐demographics, geography, local government teams and public health priorities. The recruitment of public partners who were local to each study and had some experience of the health outcome and/or public health intervention at its focus was intended to support Insight researchers to develop this understanding and design and inform the development and implementation of the studies. Researchers could not draw on existing relationships and networks with public health commissioners, practitioners and community partners.

We've argued a lot for trying to have PHIRST teams working more geographically, just to achieve those kinds of economies and to build on pre‐existing knowledge and networks, but we've never been able to persuade the NIHR of the merits of that case.

(Researcher)

Although there was variation across studies, interventions were often up and running before PHIRST teams became involved, meaning that key decisions had already been made, often without the involvement of public partners.

I think that's fairly common to all PHIRST projects. We ask, “Has there been any public involvement in the development of this intervention?” and quite often it's a no.

(Researcher)

I think it's quite difficult for the projects because often, a local authority has not done any public involvement when they're putting in the EOI [Expression of Interest].

(Researcher)

Whilst researchers believed that local authorities were supportive of public involvement, they highlighted that the level of public involvement within PHIRST Insight studies was different to the ways in which local governments were familiar with engaging members of the public.

I mean that's always something that we struggle with, I guess is kind of public participation in the planning process.

(Local Government partner)

Furthermore, it was found that the terminology used to describe public involvement differed between academics and local authorities (e.g., defining public consultations as public involvement), therefore, clarification was often required.

Researchers thought it would be better to involve public partners in intervention development, and before Expressions of Interest for PHIRST support were submitted by the local government. Because this was usually not the case, researchers had to facilitate the involvement of public partners at pace during the initial 12‐week protocol development period, meaning that sometimes public involvement could feel tokenistic, rather than an integral component embedded across the research cycle.

There are some projects where, yes, it's great if you can do a full coproduction right from the start when you're forming your ideas. But then other kinds of projects are a bit more determined if you've just got one intervention and you know from an evaluation point of view what you need to do. Then it's not that PPI involvement isn't useful, it's just useful in a different way.

(Researcher)

Some public partners also highlighted that it was not always appropriate, valuable or possible for them to be involved in certain decisions or activities throughout the studies, due to the requirements of the project and their own skill sets.

I mean I'm not sure how much I contribute to those meetings because I tend to only be able to contribute about the more general things, rather than the quite specific research type stuff.

(Public partner)

### 3.2 The Role of Public Partners and Training

In almost all of the 10 studies, a public contributor attended meetings of the study management group, a (usually) monthly meeting with the researchers and local government partners involved, set up to co‐design the study protocol, and then oversee its implementation. The group also oversaw the production of study outputs and their dissemination. Whilst it was written within the PHIRST Insight PCIEP Strategy that everyone involved would receive relevant training, it was felt that this had been limited for both public partners and academics, with several public partners stating they had not received any training.

No, I did not receive any training. And it would have been useful, definitely, to have training before we started, yes.

(Public partner)

Some researchers felt that formal training was not particularly necessary for either researchers or public partners, because of the nature of the studies and the activities public partners were asked to be involved in. However, several public contributors noted that attending study management meetings could be daunting, as they did not feel ‘expert' in research, unsure how they could best contribute and if their views would be listened to. Conversely, there were some public contributors who felt they held the balance of power, perceiving that researchers would want to defer to them.

Sometimes, I wonder if people listen too much to things that PCIEP people say. I mean, sometimes, people go a bit overboard and think, “Well, if PCIEP people are saying it, it must be right,” which suits me, but I'm not sure it's always the case.

(Public partner)

Involving the public in tasks such as developing accessible and relevant recruitment materials and data collection tools was not felt to require formal training, but rather careful explanation by the research team of the purpose of the task.

Because what we were doing wasn't that out of the box. (Researcher)

We weren't asking them to do any analysis or data collection.

(Researcher)

There were three examples where public partners were involved in data collection or analysis and were given training by members of the research team before undertaking these tasks. In one school‐based study, students were trained as peer researchers to undertake observations of school mealtimes. In two studies, public partners were involved in qualitative analysis (developing a thematic framework). However, the level of public involvement in these sorts of tasks was far less common across the 10 Insight studies than involvement in the development and review of recruitment and data collection tools. There was some concern from researchers around whether it is appropriate or not to involve public partners in data collection or analysis.

Some public members really want to be involved in analysis, and that's fine, but they're not trained as researchers. So, that's quite a big ask. I think how we handle that can be difficult.

(Researcher)

Because of the varying nature of the tasks public partners were asked to contribute to across the studies, it was evident that setting and communicating clear expectations about roles and responsibilities at the very start of the studies was important, but something which could have been done better.

I think it's about expectations on both sides, in terms of what we're expecting them to do, what they're expecting us to do, what their level of interest and engagement is. Some people really want to be fully involved. Some people don't.

(Researcher)

I think it needs to be a clear specification, in plain English, about what you expect, what you can offer in the way of support for a public involvement member, and what you expect from the public involvement member as well.

(Public partner)

It was acknowledged that a standardised training programme would not be appropriate, and instead a flexible and adaptable approach should be used, depending on the tasks public partners would be asked to contribute, the availability and needs of public partners, as well as accounting for their previous experience and skills.

I think it's different for each project, and the background and skills of each team member.

(Researcher)

There were considerations about the amount of research experience public partners have; whilst coming with prior experience was helpful, there was concern amongst both researchers and local government partners that once partners became too experienced in working with research, they might no longer reflect the views and understanding of the wider public. Both groups tended to value public members' knowledge and experience of the local area, health condition or intervention at the heart of the study, more than any previous experience of involvement in research.

Experience of being involved within the research projects or setting is helpful, but sometimes maybe it's not as helpful, because it's not more of a raw public involvement input.

(Researcher)

I'm not entirely sure that the people they ended up recruiting, they're not completely lay members of the public…. []…We know the challenges of that, and who are the members of the public, who will want to be involved, is not necessarily a fully representative sample. And how do you get a representative is the biggest question, which I think everybody asks.

(Local Government Partner)

Some respondents from both the researcher and public contributor group believed that both the representation and training concerns might be at least partially resolved by involving public members of the PHIRST management group in mentoring new public partners at the start of each Insight study. Public members of the management group tended to have more experience with public involvement activity than locally recruited contributors, and an overview of PHIRST Insight's work that they could use to support new public members. This may also address any perceived power imbalance and build confidence amongst public members to challenge researchers when they disagree about any aspect of the study.

But what we can do is offer that support, that nurture, that buddying support. What I mean by mentoring and buddying is having that one‐to‐one relationship with someone…[]…And it's just to give that level of confidence to the new member that their contribution is going to be meaningful.

(Public member)

I think there is a need for a kind of induction as what it is to be a PPI member on a Study Management Group, because at the moment it's primarily done by me…[]… You're not the PPI person's boss, but there is still a power dynamic to be considered, especially when they're in these management groups with people who are professionals in their field. So, I think having a mentor or, kind of, a general introduction about what it is to be PPI, and how to use your voice in full, might well be useful.

(Researcher)

At the beginning of PHIRST Insight, public involvement in studies was the responsibility of research leads; however, within the last year, a PCIEP manager specifically designated to support PCIEP was employed. The role of the PCIEP manager includes facilitating and supporting public involvement activities both at the managerial level of PHRST, and across individual studies. Early outputs from this new role included developing ‘onboarding' materials for new public partners explaining the PHIRST scheme, general principles of public health research, and the roles and expectations of public partners and researchers (these would also be refined and specific to individual studies). Having a PCIEP manager (instead of a researcher) as a lead contact point for public members is also intended to help address any perceived power imbalance and support public partners to make their voices heard. This new role was something valued by both public partners and researchers.

What I like about our PHIRST team now is it's in place at all levels. So, different people take responsibility for public involvement at different levels, both public contributors, but also members of the research team, so having people in place like [name of PCIEP manager], who've got an overarching thing, but then also within specific projects, within specific governance groups. So, it's a shared thing, rather than just one person's responsibility. (Researcher)

### 3.3 Impact of Public Involvement

There was a consensus among researchers that public involvement was an important part of the work of PHIRST Insight, which had evolved and improved over the 5 years since its initiation.

It's easy sometimes to feel negative about not doing public involvement well enough, but it's embedded at so many different points and so it's easy to forget about all the public involvement and engagement throughout the chain. It's good to stand back and look at everything that's going on. It's improved over the last 5‐years, but there's always more that could be done.

(Researcher)

Researchers were able to clearly highlight the value and impact that public partners had on informing studies across PHIRST. Both they and local government partners highlighted that involving the public had helped provide reassurance that the research was useful, relevant and of interest to members of the public, ensuring that elements of the study met the needs of the population group and facilitated different perspectives.

I think it has definitely given another perspective of the actual people that are taking part.

(Local Government Partner)

PHIRST researchers were able to recount a range of ways in which public involvement has impacted studies, including the development and refinement of research aims. Public members frequently supported the development of research materials, ensuring the language and terminology used were less academic and more accessible. This was the most salient impact reported by researchers.

We very much rely on our public members to try and ensure that we are using language and explaining concepts which we might take for granted, and really recognising that that may not be a thing that someone understands in another community.

(Researcher)

They particularly provided some useful feedback around, like, terminology and how to approach malnutrition, as potentially quite a sensitive topic, particularly thinking about things around frailty and age.

(Researcher)

Public partners were also able to provide useful suggestions about how to recruit participants for studies, as well as where to disseminate findings. There were instances where public partners challenged researchers' own biases.

I cycle in a busy city and [the study was about] cycling in a market town, I was thinking, “This is amazing. There's hardly any traffic. It's flat. What is everyone's problem?” So, he pulled me up on that. If you're cycling to work as a commuter and you've got places to go, actually it's just as difficult as any other place. So, that was really helpful, challenging me in the way I thought about the project…[]…my own bias.

(Researcher)

There were also examples of where public involvement throughout the duration of the research study had left a lasting impact on local government practice, such as planning to continue integrating the types of public involvement approaches used within PHIRST Insight in their future work.

Just recently, we've built a new website for our [Organisation] and wider [Organisation]. And it was something, when we were doing that, I was like, “You know, we should actually show this to older people, to the people that are going to be using this website”…. Which I don't think is something that I would have thought about before. So it is something that crosses my mind more.

(Local Government Partner)

However, some local government and public partners found it difficult to recount how impactful public involvement had been. This was in part due to a failure to routinely record the contributions of public partners and how (or if) these contributions had made a difference to the conduct of the studies. Furthermore, even where the research team was aware of the impact, this had not been shared with local government or public partners. Many public partners were unable to pinpoint tangible changes that their involvement resulted in. As a result of this work, it was identified that the recording of public involvement should be improved, with feedback to partners becoming more formalised. The PHIRST academic team has set up systems intended to facilitate more effective planning, recording, and reporting of public involvement (using an adapted version of the PIRIT toolkit [29]), with an emphasis on how impact is fed back to public partners, which will be reviewed regularly by the PCIEP manager.

Respondents from all three groups spoke about the benefits to public partners, which included feeling that their involvement allowed the public voice to be heard, with the opportunity to make improvements to their local area and develop local connections and wider networks. Public partners reported a range of personal benefits of involvement, including satisfying their own interests in the topic, personal development, and finding involvement enjoyable and stimulating.

I think it was just nice to be involved in something like that, particularly as it was a new project, something that I'd had an interest in. Watching it develop and watching seeing the benefits that people were gaining.

(Public partner)

I've learnt lots about myself, but also what my strengths are, what my weaknesses are, how I participate, what I can improve on, how I can get involved, whether my opinions are valid, whether they're relevant.

(Public partner)

## 4 Discussion

This study highlights some of the challenges and opportunities of embedding public involvement when evaluating public health interventions in collaboration with local authorities in the UK within the PHIRST scheme. Embedding public involvement is made more challenging by studies being in geographically dispersed locations, the absence of any public involvement in the development of local government interventions, and the quick timescale required for the development of study protocols. In addition, in many cases, PHIRST researchers were unfamiliar with the local context and unable to draw on existing relationships with public partners. PHIRST Insight has advocated to the NIHR that, where possible, allocating studies with better consideration of the geographies of PHIRST teams would reduce the burden for research teams and local government and public partners in scoping and developing research protocols and overseeing their implementation.

The recruitment of public partners local to the area and with some involvement with the interventions being evaluated was intended to support PHIRST researchers to develop a better understanding of the local context. Researchers' most frequently cited aim of public involvement activity was to enhance the relevance of research questions, and improve the quality of recruitment materials, data collection tools and research outputs. In this sense, public partners were valued by researchers for their ‘lay' perspective and were not required to have expertise in research. Nevertheless, it is also apparent that public partners would have benefited from better support and training. The findings from this study support the need for an agile and responsive approach to public involvement, dependent on the stages of the research cycle, delivering relevant training when required, albeit with less formal approaches which may not always be identified as ‘training’ [9].

Both public partners and researchers in this study recognised that it was not always appropriate for public partners to be involved at every stage of the research cycle. Within this study, some researchers highlighted that public partners did not have adequate expertise to be involved in data collection or analysis, and whilst this is a sentiment expressed by other authors, this is still an area of contention [11, 19]. The involvement of peers in qualitative data collection may improve the quality and validity of the data, particularly if the respondents are underserved groups [30]. It is becoming increasingly common for public partners to be involved in data analysis, supporting the generation of ‘common sense' or ‘so what' analytical approaches [19]. There were some examples of involvement in data collection and analysis in PHIRST Insight studies, but these were not common, with researchers believing in some instances that this would not have been appropriate, possible and/or useful. The degree to which public partners are involved in these aspects is always likely to vary across PHIRST studies. The quick timescale for the development and implementation of PHIRST studies also means that the level of public involvement and resources allocated to it is unlikely to evolve during individual studies, but will need to be defined as research protocols are developed. What matters is that an early consensus should be reached between researchers, local government partners and public partners regarding whether public partners should be involved in, and given adequate training and support, to contribute to some of the aspects of research that require specialist skills.

Some public members reported a lack of confidence speaking up in study management meetings with ‘expert' researchers present, a phenomenon also reported elsewhere [2, 31]. There is evidence in this study of researchers' and local government partners' wariness of the ‘paradox of representation', where training in research and experience of public involvement can professionalise public partners and diminish their lay perspective [20]. However, public partners are likely to benefit from training and support to understand research processes, including research governance and terminology, to effectively engage in discussions about research aims and contribute to the design of research tools, and can be given this support without challenge to their valued lived experience [2, 30].

As acknowledged by public partners in this study, it may be beneficial for less experienced partners to be matched up with a more experienced public member. Support from peers has been identified as beneficial in other studies of public involvement in health research [9], and PHIRST Insight will be trialling this approach by encouraging experienced public members of the Insight Management group to mentor new public members at the start of studies. Our findings also support the need to ensure there is agreement about the expectations and responsibilities of researchers, local government partners and public partners at the start of each study. This aligns with the development of a better onboarding process and provision of an induction pack to support new public partners, something which is now integrated in PHIRST Insight. The employment of a PCIEP manager in the final year of the first 5‐year PHIRST cycle was also intended to improve Insight's public involvement practices and better support public partners. Public involvement ‘leads' or ‘managers' are an emerging profession in health and social care research, but the role has been criticised as often ill‐defined [14]. Roles can range from senior leadership, leading and advising on public involvement practice, or providing administrative support (e.g., developing payment systems). However, too often they encompass both, with much of the work going unrecognised and undervalued [14]. While the employment of the PCIEP manager was valued by PHIRST researchers and public members, whether the roles and responsibilities ascribed to the post are sufficient to influence better involvement practices will need to be evaluated.

Researchers interviewed for this study placed a high value on public involvement and cited a range of impacts it had, including increasing the utility and relevance of studies for target populations, the development of better participant recruitment materials and data collection tools, challenging researchers' biases and supporting the dissemination of outputs. It is important that research teams are positive about receiving input from public partners [17], and it is encouraging that both academic researchers and local government partners felt that everyone involved in the studies was supportive of public involvement. It can be challenging to navigate different stakeholders' perspectives on what involvement should look like [32] and in this study, we did find some public partners who felt their views were *too* influential with researchers and other professionals eager to defer to them. There was also some evidence of PHIRST Insight studies influencing improved public involvement practice in the future work of local government staff.

Public partners were able to recount benefits of involvement also seen in other studies, including satisfaction in being involved in studies that aligned with their own interests [2, 33], and the development of new skills [2, 34]. However, the lack of feedback from researchers meant that many public members were unable to identify what tangible impact they had made on PHIRST Insight studies. This lack of consistent feedback has been seen in other studies and may deter public members from getting involved in future research [31]. Practical changes resulting from this work include adapting a pragmatic set of tools to support researchers working with public contributors (the PIRIT tools) [29], to facilitate more effective planning, recording and reporting of public involvement, with an emphasis on how impact is fed back to public partners (adopting a ‘you said, we did' approach). These tools are also intended to allow future studies to build on successful involvement practice, learning from previous challenges and sharing best practices across studies. Like the PCIEP manager role, the impact of these tools on PHIRST Insight public involvement practice will need to be evaluated.

### 4.1 Strengths and Limitations

To our knowledge, this is one of the first studies to explore the views of researchers, public partners and local government partners about public involvement across the same public health studies. This is important as existing research discussing the challenges of embedding involvement has typically focused on the experience of academics [14].

Whilst this study positively contributes to this field, several limitations should be acknowledged. Firstly, it was not possible to interview public partners from every PHIRST Insight study, particularly those involved in school‐based studies, or at the beginning of the 5‐year cycle, due to challenges with recontacting individuals. As a result, there is an imbalance in public member representation across studies. It is also a limitation that we did not collect demographic data about the researchers and local government staff, to allow comparison between the three groups.

Given the 5‐year span of PHIRST Insight, some participants found it difficult to recall all details within a study, particularly those involved in the initial studies. Whilst this may have limited some of the specificity given in responses, most individuals were able to critically reflect on the evolution of public involvement practices over time. It is also important to acknowledge the possibility of response bias; those who had positive experiences, or who were still involved in PHIRST Insight, may have been more likely or willing to participate, which may have skewed the perspectives given. However, this study also included contributors who had chosen to leave PHIRST Insight for a variety of reasons and were able to offer critical insights and valuable reflections based on their experiences.

Finally, whilst 67% of the public contributor interviewees are retired, this reflects a wider trend in PCIEP; this also highlights the need for more inclusive, diverse and representative recruitment, as highlighted within the wider literature [23, 35]. Indeed, the different groups involved in this study expressed a desire to improve equality, diversity and inclusion, reaching younger individuals and underrepresented groups, recognising the importance of broadening participation and facilitating more inclusive research. Ensuring diversity and equity of opportunity has been highlighted as an important component of public involvement [17, 19], and this is something that can be improved within PHIRST Insight and research more broadly.

## 5 Conclusion

Our work demonstrates the challenges of integrating public involvement throughout responsive evaluations of public health interventions embedded in local authorities. We identify that recruiting public members local to the interventions being evaluated is highly valued by researchers and local government partners. However, with many of these public members new to involvement with research, we also identify the need for appropriate training and support, including peer mentoring. We identify how our public involvement systems and practice continues to evolve, including through the introduction of a new PCIEP manager role, and improved processes for recording and feeding back on the impact of public involvement. Our findings reinforce the importance of continued reflection, resource investment, and structured processes to ensure public involvement is implemented, developed and sustained over time.

## Author Contributions

**Georgina Kathryn Wort:** investigation, writing – original draft, methodology, validation, writing – review and editing, formal analysis, data curation, project administration. **Hannah Robinson:** investigation, writing – review and editing, methodology, formal analysis, data curation, project administration. **Patricia Jessiman:** conceptualisation, investigation, methodology, validation, writing – review and editing, formal analysis, project administration, supervision. **Chloe Forte:** investigation, review and editing. **Rona Campbell:** conceptualisation, methodology, supervision, writing – review and editing. **Jemma Hawkins:** investigation, writing – review and editing. **Hannah Littlecott:** investigation, review and editing.

## Ethics Statement

This study was approved by the University of Bristol's Faculty of Health Sciences Research Ethics Committee (ref: 17879).

## Consent

All participants in this study gave written consent for their anonymised data to be used in the production of this paper.

## Conflicts of Interest

The authors declare no conflicts of interest.

## Supporting information


**Table 1:** Topic Guide for semi‐structures interviews with PCIEP partners. **Table 2:** Topic Guide for semi‐structures interviews with academic researchers. **Table 3:** Topic Guide for semi‐structures interviews with local government partners.

## Data Availability

Data supporting this study are available from the corresponding author upon reasonable request.

## References

[1] E. Agyei‐Manu , N. Atkins , B. Lee , et al., “The Benefits, Challenges, and Best Practice for Patient and Public Involvement in Evidence Synthesis: A Systematic Review and Thematic Synthesis,” Health Expectations 26, no. 4 (August 2023): 1436–1452, 10.1111/hex.13787. 37260191 PMC10349234

[2] J. Brett , S. Staniszewska , C. Mockford , et al., “A Systematic Review of the Impact of Patient and Public Involvement on Service Users, Researchers and Communities,” Patient‐Patient‐Centered Outcomes Research 7, no. 4 (December 2014): 387–395. 25034612 10.1007/s40271-014-0065-0

[3] C. McGrath , G. Lasseter , N. Hopewell‐Kelly , et al., “How Do We Get the Public Into Public Health Research? Learnings and Key Recommendations From Initiating a Community Involvement Project Scheme,” Health Expectations 27, no. 6 (December 2024): e70114, 10.1111/hex.70114. 39648476 PMC11625873

[4] J. Boote , R. Wong , and A. Booth , “‘Talking the Talk or Walking the Walk?’ A Bibliometric Review of the Literature on Public Involvement in Health Research Published Between 1995 and 2009,” Health Expectations 18, no. 1 (February 2015): 44–57, 10.1111/hex.12007. 23033933 PMC5060762

[5] NHS Health Research Authority , The Shared Commitment to Public Involvement in Health and Social Care Research, January 2026, https://www.hra.nhs.uk/planning-and-improving-research/best-practice/public-involvement/shared-commitment-public-involvement-health-and-social-care-research/.

[6] O. R. Phillips , C. Harries , J. Leonardi‐Bee , et al., “What Are the Strengths and Limitations to Utilising Creative Methods in Public and Patient Involvement in Health and Social Care Research? A Qualitative Systematic Review,” Research Involvement and Engagement 10, no. 1 (May 2024): 48, 10.1186/s40900-024-00580-4. 38741156 PMC11092192

[7] H. J. Westerink , T. Oirbans , M. M. Garvelink , et al., “Barriers and Facilitators of Meaningful Patient Participation at the Collective Level in Healthcare Organizations: A Systematic Review,” Health Policy 138 (December 2023): 104946, 10.1016/j.healthpol.2023.104946. 38000333

[8] D. W. Smits , K. Van Meeteren , M. Klem , M. Alsem , and M. Ketelaar , “Designing a Tool to Support Patient and Public Involvement in Research Projects: The Involvement Matrix,” Research Involvement and Engagement 6, no. 1 (June 2020): 30, 10.1186/s40900-020-00188-4. 32550002 PMC7296703

[9] O. L. Aiyegbusi , C. McMullan , S. E. Hughes , et al., “Considerations for Patient and Public Involvement and Engagement in Health Research,” Nature Medicine 29, no. 8 (August 2023): 1922–1929, 10.1038/s41591-023-02445-x. 37474660

[10] S. Oliver , K. Liabo , R. Stewart , and R. Rees , “Public Involvement in Research: Making Sense of the Diversity,” Journal of Health Services Research & Policy 20, no. 1 (January 2015): 45–51, 10.1177/1355819614551848. 25228453

[11] J. Russell , T. Greenhalgh , and M. Taylor , Patient and Public Involvement in NIHR Research 2006–2019: Policy Intentions, Progress and Themes. National Institute for Health Research: Oxford, UK, 2019 Feb.

[12] National Health Service Act , S13Q, 2006, https://www.legislation.gov.uk/ukpga/2006/41/section/13Q.

[13] J. Ocloo , S. Garfield , B. D. Franklin , and S. Dawson , “Exploring the Theory, Barriers and Enablers for Patient and Public Involvement Across Health, Social Care and Patient Safety: A Systematic Review of Reviews,” Health Research Policy and Systems 19, no. 1 (January 2021): 8, 10.1186/s12961-020-00644-3. 33472647 PMC7816359

[14] S. Papoulias and L. M. Brady , “I Am There Just to Get on With It”: A Qualitative Study on the Labour of the Patient and Public Involvement Workforce,” Health Research Policy and Systems 22, no. 1 (September 2024): 118, 10.1186/s12961-024-01197-5. 39223597 PMC11367993

[15] R. Pandya‐Wood , D. S. Barron , and J. Elliott , “A Framework for Public Involvement at the Design Stage of NHS Health and Social Care Research: Time to Develop Ethically Conscious Standards,” Research Involvement and Engagement 3, no. 1 (April 2017): 6, 10.1186/s40900-017-0058-y. 29062531 PMC5611655

[16] S. Staniszewska , C. Mockford , A. Gibson , S. Herron‐Marx , and R. Putz , “Moving Forward: Understanding the Negative Experiences and Impacts of Patient and Public Involvement in Health Service Planning, Development and Evaluation,” in Critical Perspectives on User Involvement, ed. M. Barnes (Policy Press, 2011), 129–141, 10.51952/9781847429483.ch011.

[17] P. Wilson , E. Mathie , J. Keenan , et al., “ReseArch With Patient and Public invOlvement: A RealisT Evaluation‐the RAPPORT Study,” Health Services and Delivery Research 3, no. 38 (2015): 1–176, 10.3310/hsdr03380. 26378332

[18] J. C. Crocker , I. Ricci‐Cabello , A. Parker , et al., “Impact of Patient and Public Involvement on Enrolment and Retention in Clinical Trials: Systematic Review and Meta‐Analysis,” BMJ (Clinical Research Ed.) 363 (November 2018): 4738, 10.1136/bmj.k4738. PMC6259046 30487232

[19] P. Hoddinott , A. Pollock , A. O'Cathain , et al., “How to Incorporate Patient and Public Perspectives Into the Design and Conduct of Research,” F1000Research 7 (2018 Jun 18): 752. 30364075 10.12688/f1000research.15162.1 PMC6192439

[20] J. Ives , S. Damery , and S. Redwod , “PPI, Paradoxes and Plato: Who's Sailing the Ship?,” Journal of Medical Ethics 39, no. 3 (March 2013): 181–185, 10.1136/medethics-2011-100150. 22267385

[21] M. Cullen , C. Cadogan , S. George , et al., “Key Stakeholders' Views, Experiences and Expectations of Patient and Public Involvement in Healthcare Professions' Education: A Qualitative Study,” BMC Medical Education 22, no. 1 (April 2022): 305, 10.1186/s12909-022-03373-z. 35449105 PMC9026974

[22] S. E. Knowles , P. Walkington , J. Flynn , S. Darley , R. Boaden , and R. Kislov , “Contributors Are Representative, as Long as They Agree: How Confirmation Logic Overrides Effort to Achieve Synthesis in Applied Health Research,” Health Expectations 25, no. 5 (October 2022): 2405–2415, 10.1111/hex.13555. 35959510 PMC9615063

[23] National Institute for Health Research , Improving Inclusion of Under‐Served Groups in Clinical Research: Guidance From the NIHR‐INCLUDE Project. UK: NIHR, 2020, www.nihr.ac.uk/documents/improving-inclusion-of-under-served-groups-in-clinical-research-guidance-from-include-project/25435.

[24] National Institute for Health Research , Public Health Intervention Responsive Studies Team. n.d., https://phirst.nihr.ac.uk/.

[25] National Institute for Health Research , Public Health Intervention Responsive Studies Teams (PHIRST) ‐ Process and Outcomes Evaluation, 2024, https://www.nihr.ac.uk/funding/public-health-intervention-responsive-studies-teams-phirst-process-and-outcomes-evaluation/2464#tab-367666.

[26] National Institute for Health Research , PHIRST Insight, July 21, 2025, https://phirst.nihr.ac.uk/about-phirst/phirst-insight/.

[27] National Institute for Health Research , Public Health Intervention Responsive Studies Team: PHIRST Insight, 2021, https://fundingawards.nihr.ac.uk/award/NIHR131567.

[28] N. K. Gale , G. Heath , E. Cameron , S. Rashid , and S. Redwood , “Using the Framework Method for the Analysis of Qualitative Data in Multi‐Disciplinary Health Research,” BMC Medical Research Methodology 13, no. 1 (September 2013): 117, 10.1186/1471-2288-13-117. 24047204 PMC3848812

[29] M. B. Newman , K. Seddon , S. Peddle , and A. Nelson Public Involvement In Research Impact Toolkit (PIRIT), 2023, https://www.cardiff.ac.uk/marie-curie-research-centre/patient-and-public-involvement/public-involvement-in-research-impact-toolkit-pirit.

[30] K. Staley , “There Is No Paradox With PPI in Research,” Journal of Medical Ethics 39, no. 3 (March 2013): 186–187, 10.1136/medethics-2012-100512. 23288267

[31] A. Howe , H. MacDonald , B. Barrett , and B. Little , “Ensuring Public and Patient Participation in Research: A Case Study in Infrastructure Development in One UK Research and Development Consortium,” Primary Health Care Research & Development 7, no. 1 (January 2006): 60–67.

[32] T. Greenhalgh , L. Hinton , T. Finlay , et al., “Frameworks for Supporting Patient and Public Involvement in Research: Systematic Review and Co‐Design Pilot,” Health Expectations 22, no. 4 (August 2019): 785–801, 10.1111/hex.12888. 31012259 PMC6737756

[33] F. Ross , S. Donovan , S. Brearley , et al., “Involving Older People in Research: Methodological Issues,” Health & Social Care in the Community 13, no. 3 (2005 May): 268–275, 10.1111/j.1365-2524.2005.00560.x. 15819748

[34] A. Rowe , “The Effect of Involvement in Participatory Research on Parent Researchers in a Sure Start Programme,” Health & Social Care in the Community 14, no. 6 (November 2006): 465–473, 10.1111/j.1365-2524.2006.00632.x. 17059488

[35] D. Faluyi , P. V. Ovseiko , K. Dziedzic , et al., “NIHR Race Equality Framework: Development of a Tool for Addressing Racial Equality in Public Involvement,” Research Involvement and Engagement 10, no. 1 (May 2024): 44, 10.1186/s40900-024-00569-z. 38715152 PMC11077722

