# Supplementary material for: Public Involvement in the Evaluation of Local Government Public Health Interventions in the UK: Lessons From PHIRST Insight
Source: Health Expect. 2026 Feb 26;29(2):e70618. doi: 10.1111/hex.70618 (PMC12936984; doi:10.1111/hex.70618)
Supplement: Supplementary file 1 — Table 1: Topic Guide for semi‐structures interviews with PCIEP partners. Table 2: Topic Guide for semi‐structures interviews with academic researchers. Table 3: Topic Guide for semi‐structures interviews with local government partners. [file HEX-29-e70618-s001.docx]

## Supplementary Information: Topic Guides

Table i. Topic Guide for semi-structures interviews with PCIEP partners

| **Topic Area** | Question | - Prompt |
| --- | --- | --- |
| **Background** | Please start by telling me a little bit about yourself: | - Employment (past and present) - Voluntary roles - Caring responsibilities |
|  | Before your work with PHIRST Insight, had you ever been involved in research? | - In what role (researcher, participant, public partner, other; note if involved in more than one study, capture all) - What was the focus of the research? - When was this? |
|  | Please can you summarise which project(s) you were involved in and the types of activities you did (we will ask more detail about this later in the interview). |  |
| **Getting Involved** | How did you get involved with the PHIRST Insight study? | - *Responded to recruitment email/poster etc* - *Contacted by someone you know* - *Contacted by an organisation* - *Other* |
|  | Why did you want to get involved in the study? | - *Interested in the topic* - *Interested in research* - *Knowledge about the target population* - *Looking for a role/opportunity* - *other* |
|  | When you first became involved, were you clear what your role would be? | - *Why/why not* - *Level of information available at the time* |
|  | Is there anything about this early stage of becoming involved with a PHIRST Insight study that might be improved? |  |
| **Role on the PHIRST Insight study** | How many public partners were involved in the study | - *Just you, or more than that* - *Did you know the other public partners before this?* |
|  | Please describe your role on the PHIRST Insight study – what were the sorts of things you were involved in? | - *Study management group member* - *Part of a group of public partners that the researchers convened* - *Development of study protocol/research questions/logic model etc* - *Development of study materials e.g. recruitment materials, topic guides, survey questions etc* - *Supporting recruitment* - *Data analysis* - *Writing/reviewing study outputs* - *Attending events* - *Supporting PCIEP monitoring (e.g. the PIRIT tool)* - *other* |
|  | Did you understand *why* you were involved in the study?  Are these the sorts of things you expected to be involved in?  Did your role change over time? How did this come about? |  |
|  | How did you find these tasks? | - *Easy/challenging and why* - *Interesting/boring* |
| *Share screen and fill in radar plot (Figure 1) with them based on their thoughts of PCIEP partners’ involvement at the different stages of the project– clarify the different levels of involvement as needed and answer any questions* | | |
|  | Is there anything else you would have liked to have been involved in (but weren’t)?  (If appropriate) Do you think you had the same opportunities to contribute as other public partners involved with the project? | If not, explore |
|  | Did you receive any training from or organised by the research team during your time on the study? | - *If yes, details and usefulness* - *If no, would any training have been useful?* |
|  | Did you receive other types of support that helped you contribute? Is there other support you would have liked that you didn’t receive? | - *Prompt for support from peers or more experienced public partners* - *IT support* |
|  | Was the language used in meetings and in communications appropriate?  What would you say was your main influence on the study? | - *Did anything change/was done differently because of your involvement? How do you know this?* - *Would you have liked to have had more influence? In what way?* - *Was your influence on the study recorded in any way?* |
|  | Who was your main contact on the study team?  How often did you meet? | - *In person/online/mix* - *Was this convenient for you?* - *Was the duration of meetings reasonable or burdensome* |
|  | Do you think that the research team listened to you?  Do you think they valued your input and opinion? | - *Were the research team open to change and suggestions?* - *Why do you say this* - *Did you experience a hierarchy or any power imbalances between the research team and public partners?* - *Explore any difference between researchers, local authority staff, others and their attitude to public partners* |
|  | Could anything be improved/done differently that would have made your experience better? |  |
| **Impact of involvement** | Did you enjoy being involved with the study? |  |
|  | Has being involved had any advantages for you? Any disadvantages? | - *Skills development* - *Learning new knowledge* - *Meeting new people* - *Influencing decisions or making a difference* - *Creating other opportunities* - *Other* |
|  | Did your involvement change your approach to your own health or wellbeing in any way?  Were there any barriers to you engaging with the study team or becoming more involved? |  |
|  | Were you paid for your time on the study? Did you receive any form of recognition for your involvement | - *Was payment reasonable recompense for time spent* - *Did you incur any expenses that were not reimbursed* - *Certificate/letter etc* - *What kind of recognition would be meaningful for you?* |
|  | Would you like to be involved in research as a public partner again? | - *Why do you say this* - *Would you recommend it to others* - *Would you like to be involved if possible in future?* |
|  | Did you have the chance to feedback to the team about your experiences of involvement? | - *Did anything change as a result?* - *Would this be welcome if it didn’t happen?* - *When / what form of feedback?* |
|  | Is there anything else you would like to tell us about your experience of being involved? |  |
|  | Did you managed to fill out the demographic questionnaire okay? How would you like to be paid for today? |  |

Table ii. Topic Guide for semi-structures interviews with academic researchers

| **Topic Area** | Question | Prompt |
| --- | --- | --- |
| **Background** | What project(s) were you involved in? [for the purpose of the recording] |  |
|  | Were you involved throughout the different stages of research? |  |
|  | What was your role on the study team, and what were your main responsibilities on the project? |  |
| **PCIEP Involved** | Could you give us a summary of the public involvement in this project? Who was involved and how?  What was their level of involvement in the different stages of the project?  Was their involvement one-time or continuous? | - Developing protocol - Recruitment - Designing data collection tools - Data collection - Data analysis - Producing outputs - Dissemination |
|  | How were roles and responsibilities decided at each stage, for both PCIEP partners and researchers? |  |
|  | Who was the main contact for PCIEP partners? | - How was this decided? - What do you think makes a good PCIEP lead? - Were there any conflicts? How were these managed? - Were all stakeholders/ those involved in the project aligned on the focus and outcome of the project? |
| - *Share screen and fill in radar plot (Figure 1) with them based on their thoughts of PCIEP partners’ involvement at the different stages of the project– clarify the different levels of involvement as needed and answer any questions* | | |
|  | Were any framework/models used when planning public involvement in the project? | - *Piirt tool, involvement matrix* |
|  | Had any PCIEP work been undertaken by the local authority/other local partners before PHIRST Insight became involved? | - *If yes, describe* - *Was this useful in setting up PCIEP for the evaluation* - *If no, why not* |
|  | Alongside public involvement activities, were there any public engagement activities /knowledge exchange activities in this project? |  |
|  | Were local authority/other local partners/ researchers supportive of public involvement in the evaluation? | - *Did any of them know each other before joining the project?* - *Who introduced the project and explained PCIEP involvement?* - *Were barriers/facilitators to inclusive participation considered?* - *What socio-demographic information was collected from PCIEP partners?* - *Were there any challenges in collecting this information?* - *Do you think participants were ‘representative’ of the wider community/population of interest?* |
|  | How were PCIEP partners recruited? |  |
|  | Was training given at any of these stages (for public partners and/or researchers)? If so, what? | - Was this training organised as part of the PHIRST project, or had public partners had any previous experience or exposure to training? Do you know who provided/ how they accessed it? |
|  | What resources were important at the different stages? Is there anything else you'd have liked/needed? |  |
|  | Were health inequalities considered within this project? Or considerations for different groups? |  |
|  | Was there any flexibility in PCIEP involvement at any stage? If so, what? | - Were there any adaptions for context? |
|  | Was language considered at any stage? If so how? Were any public partners involved in these decisions? |  |
|  | Were power dynamics considered? Do you think these dynamics influenced the project and PCIEP involvement in any way? |  |
|  | What were the location and settings for PCIEP? | - E.g. remote/online; if face to face what venues were used and suitability - Considerations for fostering an inclusive and collaborative environment? |
|  | Did any ethical issues/ challenges arise at any point? | - *Were there any safeguarding concerns?* - *Did PCIEP partners need DBS checks?* |
|  | What benefits were there for public partners being involved? | - *i.e., other than being paid did they accumulate any other skills, food...* |
| **Impact of PCIEP** | Do you think public involvement worked well in this study, or not? |  |
|  | What difference did public involvement make? | - *Was public involvement logged/tracked throughout the study? How?* |
|  | What do you think wider staff and PCIEP partners views towards PCIEP were? |  |
|  | What would have helped improve PCIEP involvement?    What would you implement again? |  |
| **Partnership with LA(s)** | What is your overall reflection of working with the local authority(s) involved in the evaluation?  Did you feel you had enough opportunity to understand the local context of the intervention? | - *The LA’s reasons for running this intervention* - *Their reasons for wanting the study* - *Political context* - *Financial context* - *Regulatory issues* - *Local partnerships (e.g. with organisations delivering the intervention)* - *Their preferred timescales/deadlines* - *Other local factors* - *(If no opportunity to understand context, why not)* |
|  | What was the relationship like between the research team and colleagues in the local authority(s)? | - *Trust/respect* - *Any power imbalance* - *Nature of meetings – frequency, online/face to face – experience of these* - *Language barriers and/or shared understanding of terms* - *Where the relationship worked well, what enabled this?* - *Any improvements/changes that you would suggest* |
|  | Thinking about co-production, (meaning the involvement of LAs and other local stakeholders in collaborating and shared decision making in all aspects of the study), what was your experience of this? | - *Who was involved in the development of the research protocol?* - *How were these people identified? Were they the ‘right’ people?* - *Who was involved in developing…* - *The logic model* - *The research questions* - *The study design and methods* - *Agreeing/producing the outputs of the study (publications, reports, infographics etc)* - *Any changes (to protocol, outputs, dissemination plans)* - *How do feel you this this process went?* - Any benefits of co-production? Disadvantages? - What would have improved this process? |
|  | Aside from the local authority(s) involved, were other organisations involved in the evaluation? If so, how? E.g. third party organisations, health care professionals?  Q/ Did working with these organisations present any additional challenges in the evaluation process? E.g. data sharing agreements |  |
|  | Anything else? |  |

Table iii. Topic Guide for semi-structures interviews with local government partners

| **Topic Area** | Question | Prompt |
| --- | --- | --- |
| **Background** | Current role and responsibilities | - Are these the same as when working on the PHIRST Insight study? |
|  | Previous experience of conducting/commissioning research or evaluation as part of role  Previous experience of working with academic researchers | - And other research organisation(s) |
| **Applying to PHIRST** | What were the main reasons for applying to the PHIRST scheme? |  |
|  | Who in your organisation (or other) wanted to apply? (their role) |  |
|  | Was anyone else involved in this decision (DPH, elected partners, other departments/partners) |  |
|  | What information did you want the study to explore? | - Evidence of impact of the intervention - Understanding implementation – best practice - Understand users’ experience/needs - Maintenance of/securing funding - Cost effectiveness - Other evidence/information gaps - Explore whether study intended to produce local insight or to be more generalisable |
|  | Did the PHIRST Insight team understand these aims? | - Were they reflected in the research questions of the study - Explore **changes**/additions/scaling back and why. |
|  | Had you explored other options for undertaking the research? | - *In-house* - *Commissioning externally* - *Previous bids/applications or studies* - *Reasons why not/why these were unsuccessful (esp any barriers to engaging with academic researchers)* - *Would you have undertaken this study through another means if the PHIRST application had been unsuccessful?* |
|  | Did you have any other expectations of benefits working with PHIRST? | - *Reputational – being a LA that is research-ready/values research* - *Building staff capacity to engage with/interpret/undertake research* - *Other wider benefits* |
| **Working with PHIRST Insight** | Tell us your overall reflections on working with PHIRST Insight |  |
|  | Did you feel you had enough opportunity to explain the local context to the PHIRST Team? | - *Reasons for running this intervention* - *Reasons for wanting the study* - *Political context* - *Financial context* - *Regulatory issues* - *Local partnerships (e.g. with organisations delivering the intervention)* - *Your preferred timescales/deadlines* - *Other local factors* - *(If no opportunity to explain context, why not)* |
|  | Did the PHIRST team pay heed to this throughout the study? | - If yes, in what way and benefits of this - If not, how might this be improved and any consequences of not heeding local contextual factors |
|  | What was the relationship like with the PHIRST team? | - *Trust/respect* - *Any power imbalance* - *Nature of meetings – frequency, online/face to face – experience of these* - *Language barriers and/or shared understanding of terms* - *: e.g. research, impact, evidence, public involvement etc* - *Other* - *Where the relationship worked well, what enabled this?* - *Any improvements/changes that you would suggest* |
|  | Were you aware of any constraints on what the PHIRST team could offer? | - Availability/expertise of research staff - Regulatory issues – ethics; data sharing agreements etc - Funding - Timescales - Other - Impact of these on the study/relationship |
|  | Thinking about co-production, (meaning the involvement of you and other local stakeholders in collaborating and shared decision making in all aspects of the study), what was your experience of this? | - *Who was involved in the development of the research protocol?* - *How were these people identified? Were they the ‘right’ people?* - *Who was involved in developing…* - *The logic model* - *The research questions* - *The study design and methods* - *Agreeing/producing the outputs of the study (publications, reports, infographics etc)* - *Any changes (to protocol, outputs, dissemination plans)* |
|  | How do feel you this this process went? | - Did the academic team ‘lead’? Was that appropriate (or not) - Did you/others have sufficient influence? Shared decision-making? - Any benefits of co-production? Disadvantages? - What would have improved this process/helped you stay more actively involved? - Was it what you expected when you applied to PHIRST? |
|  | And thinking about public involvement, (meaning the involvement of local partners of the public who are affected by the intervention in the study design, delivery and outputs) | - *What was your understanding of public involvement at the time you applied to PHIRST? What did this term mean to you?* - *Had any public involvement work happened to inform the intervention before PHIRST involvement?* - *What, and why/why not* - *Did the PHIRST team facilitate public involvement?* - *How, and with who* - *Influence on protocol, research questions, methods, outputs, study management group, other* |
|  | Do you think public involvement worked well in this study, or not? |  |
|  | What would have helped it be done better? |  |
|  | What difference did public involvement make? |  |
|  | Has the experience of public involvement on the PHIRST study changed anything for your local authority? | - *Changed their approach to PPI* - *Encouraged/discouraged PPI in local practice* - *Impacted local relationships (with public partners, organisations, other)* |
| **Impact** | What is the status of the intervention now? (e.g. still ongoing, stopped, expansion, changes to implementation etc) | - *Why is this?* - *Is it what you wanted to happen?* - *Any planned changes?* - *If appropriate, is any of this a result or influenced by the PHIRST study?* |
|  | What was your response to the study findings? | - *Any unexpected/disputed findings?* - *Any that caused local concern/distress and why?* - *Positive findings – as expected? Local response to these.* |
|  | What were the main outputs of the study (reports, slide-sets, info-graphics etc) | - *Were these useful (and in what way)* - *How were they shared, and with whom* - *LA staff incl at senior level* - *Elected partners* - *Other local organisations/partners* - *Local residents/service users* - *Wider audience(s) outside the LA* - *In all cases, what was the response/feedback* - *Would any other types of output have been helpful?* |
|  | Has anything changed as a result of the study findings? | - *Implementation of the intervention (if not already covered) – should include delivery, practice, scope, scale, reach, how users experience the service etc* - *Sustainability (or not) of funding* - *Informed any funding decisions* - *Informed any local policy/strategy* - *(Prompt to share anything we can cite as impact)* - *Other* |
|  | And thinking about your organisation, has anything changed with regard to your approach to research? | - *Motivation to engage with academic research* - *Motivation/capacity to apply for other research funding* - *Understanding/capacity to undertake or commission research* - *Organisational/personal reputation re research* |
|  | Has involvement with PHIRST helped the career development of staff? | - *Learning and development* - *New (research roles) roles* - *Applied for funding/fellowships etc* |
| **Final questions** | Given everything we have discussed, do you think your involvement with the PHIRST scheme went well? What would you change? |  |
|  | Would you/have you applied again? |  |
|  | What advice would you give other local authorities considering applying for PHIRST support? |  |
|  | And what advice would you have for academic teams working with you? How could they do better? |  |
|  | Anything else you would like to add |  |
